# A Putative Non-Canonical Ras-Like GTPase from *P*. *falciparum*: Chemical Properties and Characterization of the Protein

**DOI:** 10.1371/journal.pone.0140994

**Published:** 2015-11-05

**Authors:** Annette Kaiser, Barbara Langer, Jude Przyborski, David Kersting, Mirko Krüger

**Affiliations:** 1 Medical Research Centre, Institute for Pharmacogenetics, University Duisburg-Essen, Hufelandstrasse 55, 45147 Essen, Germany; 2 Institute for Pharmacology, Hufelandstrasse 55, 45147 Essen, Germany; 3 Department of Parasitology, FB Biology, Phillipps University Marburg, Karl-von Frisch-Strasse 8, 34043 Marburg, Germany; Institut national de la santé et de la recherche médicale—Institut Cochin, FRANCE

## Abstract

During its development the malaria parasite *P*. *falciparum* has to adapt to various different environmental contexts. Key cellular mechanisms involving G-protein coupled signal transduction chains are assumed to act at these interfaces. Heterotrimeric G-proteins are absent in *Plasmodium*. We here describe the first cloning and expression of a putative, non-canonical Ras-like G protein (acronym PfG) from *Plasmodium*. PfG reveals an open reading frame of 2736 bp encoding a protein of 912 amino acids with a theoretical pI of 8.68 and a molecular weight of 108.57 kDa. Transcript levels and expression are significantly increased in the erythrocytic phase in particular during schizont and gametocyte formation. Most notably, PfG has GTP binding capacity and GTPase activity due to an EngA2 domain present in small Ras-like GTPases in a variety of Bacillus species and *Mycobacteria*. By contrast, plasmodial PfG is divergent from any human alpha-subunit. PfG was expressed in *E*. *coli* as a histidine-tagged fusion protein and was stable only for 3.5 hours. Purification was only possible under native conditions by Nickel-chelate chromatography and subsequent separation by Blue Native PAGE. Binding of a fluorescent GTP analogue BODIPY® FL guanosine 5’O-(thiotriphosphate) was determined by fluorescence emission. Mastoparan stimulated GTP binding in the presence of Mg^2+^. GTPase activity was determined colorimetrically. Activity expressed as absolute fluorescence was 50% higher for the human paralogue than the activity of the parasitic enzyme. The PfG protein is expressed in the erythrocytic stages and binds GTP after immunoprecipitation. Immunofluorescence using specific antiserum suggests that PfG localizes to the parasite cytosol. The current data suggest that the putitative, Ras-like G-protein might be involved in a non-canonical signaling pathway in *Plasmodium*. Research on the function of PfG with respect to pathogenesis and antimalarial chemotherapy is currently under way.

## Introduction

Knowledge of pathways regulated by cyclic nucleotides in *Plasmodium* is still rudimentary [1]. Both nucleotides cGMP and cAMP increase during stage conversion of the asexual, intraerythrocytic stages to the presexual stages. In 2009 the malaria signaling consortium [2] has been founded to study the molecular mechanisms which enable the parasite to sense and adapt to the intra- and extra-cellular requirements, i.e. invasion of the hepatocytes in the human liver, the erythrocytic stages in the human host and the sexual development in the *Anopheles* mosquito. These switches are a prerequisite for proliferation and transmission of *Plasmodium*. Knowledge about the first part of the pathway in which a GPCR-mediated signal might be transferred via heterotrimeric G-proteins to adenylate cyclase is still rudimentary. Adenylylcyclases [3] and guanylcyclases [4] together with their cAMP dependent-kinases have been already identified in *Plasmodium* [5]. Some of the corresponding protein kinases like CDPK4 [6], protein kinase A [7], protein kinase B [8] have been closely related with the production of male gametes. The results of these investigations are of significant relevance in accelerating the eradication by novel kinase inhibitors [9].

A major issue in search for novel pathways with potential drug targets is the discovery of eukaryotic signal transduction pathways which operate through a limited number of effectors. One widely used principle is signaling through G-protein-coupled-receptors (GPCR) [10]. GPCRs are a heterogenous group of proteins like hormones [11], pheromones [12], odorant [13] and light receptors [14]. Hitherto, no such receptors have been identified in *Plasmodium*. GPCRs transduce their external signals via heterotrimeric G-proteins to mitogen activated protein kinase dependent pathways [6,7,8,9].

However, parasite-specific endogenous, heterotrimeric G-proteins are absent in these signaling events. Although there is no genomic evidence that conserved heterotrimeric G- proteins exist in *Plasmodium*, cAMP dependent kinases [6, 7, 8, 9] have been identified suggesting at least the existence of cAMP regulated pathways in the parasite. Signaling mediated by GPCR-coupled pathways starts with the binding of a ligand to a membrane-bound G-protein-coupled receptor (GPCR). GPCRs were not identified in *Plasmodium* but four sequences are present in PlasmoDB which might encode putative GPCRs. In canonical GPCR-coupled pathways binding of a ligand leads to a conformational change in the receptor protein. Heterotrimeric G-proteins which are composed of alpha, beta and gamma subunits are triggered to interact with the receptor [13]. Once a receptor is activated the GDP which is bound to the Gα-subunit is exchanged to GTP and the Gα-subunit dissociates from the receptor and modulates downstream effectors like adenylate cyclases and phosphodiesterases. The human Gα-subunits consist of four different subfamilies [6] i.e. Gαs, Gαi/o, Gαq11 and Gα 12/13 which lead to a variety of downstream signals although only one receptor protein is present.

To understand the pathogenesis of a malaria infection, signaling processes in the human erythrocyte and presumably the parasitophorous membrane have to be considered. Recently, in the enucleated human erythrocyte an increasing number of proteins were identified which are involved in signaling [14]. For an outside-in signaling membrane receptors such as purinergic receptors are responsible [15]. Inside-out signaling is accomplished by ATP [16]. A strong stimulation of erythrocyte ion channel activity is observed after the intraerythrocytic amplification of the malaria parasite *P*. *falciparum* [17]. It has recently been shown that 12 different proteins residing in lipid rafts of the erythrocyte membrane are recruited to the parasitophorous vacuole [18]. Two of these proteins are the erythrocyte β2-adrenergic receptor and the heterotrimeric guanine nucleotide–binding protein (G- protein). Erythrocytic G- proteins reside at the cytoplasmic face of the cellular plasma membrane, where they can couple with a variety of transmembrane receptors to transduce extracellular signals to proteins (Gαs) and thus facilitate invasion of the parasite [19]. Recent results showed that the erythrocyte host G-alpha-s subunit (G_s_) is functional and promotes invasion of the parasite [20] into the erythrocyte. Supplementation of *in vitro P*. *falciparum* cultures with propranolol, an antagonist of the G protein-coupled ß-adrenergic receptor inhibited intracellular parasite growth. In sum, obstruction of signal transduction via the erythrocyte reduced invasion of the parasite [21]. These results led to the conclusion that the erythrocyte G protein might be considered as a novel target for antimalarial chemotherapy [21]. Moreover, it was concluded that signaling in *Plasmodium* is unlikely to be via parasitic, heterotrimeric G-proteins and depends exclusively on GPCR mediated signaling in the erythrocyte of the human host.

There is further evidence which supports a role for the host Gs signal transduction in severe malaria pathogenesis [22]. Different associations in the six Gs pathway candidate genes like human adenosine receptor 2A (*ADORA2A*) and 2B (*ADORA2B*), beta-adrenergic receptor kinase 1 (*ADRBRK1*), adenyl cyclase 9 (*ADCY9*), G-protein beta subunit 3 (*GNB3*), and regulator of G-protein signaling 2 (*RGS2*) have been identified. Most notably is the finding that the G allele (approximately 20% frequency) confers enhanced risk to severe malaria [22]. These results further supported the hypothesis that the malaria parasite uses the human Gs host protein for its signaling processes.

Recent results demonstrated that treatment of *P*. *falciparum in vitro* cultures with cholera toxin or mastoparan 7 increased gametogenesis [23]. While cholera toxin as a protein stimulates ADP-ribosylation of the Gs alpha-subunit [24], mastoparan 7 a peptide from wasp venom stimulates GTPase activity and promotes dissociation of any GDP bound to the receptor protein thus activating GTP binding. The experiments [21] showed that only mastoparan 7 significantly increased cAMP levels. These results led to the conclusion that cholera toxin induced increased commitment to gametogenesis but heterotrimeric G-proteins are absent. In sum, the current controversy of the data prompted us to reinvestigate these results and to screen for proteins which might substitute canonical G-proteins in *P*. *falciparum*. Surprisingly, our screening approach resulted in the identification of a non-canonical Ras-like GTPase PfG with GTP-binding and GTPase like properties confirming the absence of heterotrimeric G-proteins in *Plasmodium*.

## Material and Methods

### Isolation of subcellular RNA

Isolation of cellular RNA was performed by the Trizol® method (Invitrogen, Karlsruhe, Germany) [25]. Total cellular RNA was isolated from synchronized *P*. *falciparum in vitro* cultures containing schizonts for the cloning of *PfG*. The RNA concentration was 323 ng /μL. Subsequently isolation of poly(A)^+^ RNA (mRNA) was performed by oligo(dT) cellulose as an affinity matrix according to a protocol from Invitrogen, Mannheim, Germany. Poly(A)^+^ RNA containing fractions were pooled and the concentration was determined 0.2 μg/μL. 1.2 μg poly (A)^+^RNA was applied in RT-PCR reactions (see Material and Methods within).

### Isolation of cellular RNA from different developmental stages and determination of the stage specific transcript levels of PfG mRNA by Northern Blot analysis

For the isolation of cellular RNA from different developmental stages, synchronized *P*. *falciparum in vitro* cultures for each state and gametocyte cultures [26] were employed. Cells were lysed with a QIAshredder and extraction of cellular RNA followed subsequently according to the protocol of the RNAeasy Minikit (Qiagen, Hilden, Germany). Northern Blot analysis was performed according to the protocol of the Digoxin (Dig) High Prime DNA Labeling and Starter Kit (Roche, Ingelheim, Germany). In principle, the *PfG* mRNA was labeled with Dig-dUTP and hybridized against the cellular RNA from different developmental stages which was transferred onto a nitrocellulose membrane. Anti-digoxigenin antibody was combined with 3-(1-chloro-3'-methoxyspiro[adamantane-4,4'-dioxetane]-3'-yl)phenyl] dihydrogen phosphate (CSPD) to detect the signals after exposition on an X-ray film.The detected signals were analyzed by a phosphoimager (Molecular Dynamics, Göttingen, Germany) and expressed as % relative transcription in comparison to the constitutively transcribed control [27] and the stage specific controls [28].

### PCR amplification of the PfG coding sequence from *P. falciparum* strain NF54 by RT PCR and cloning strategy

PCR amplification by RT-PCR was performed with the RT-PCR Access KIT (Promega, (Madison, WI, USA). A final PCR volume of 50 μL contained: 33 μL of nuclease-free water, AMV⁄ Tfl 5 fold reaction buffer, 10 μL, dNTP Mix (10 mM each dNTP) 0.2 mM, upstream primer G1 5’-ATGAAAAATGTTTTTAAAAAAAGAG-3’; downstream primer G10 5’- AAGGAGTTCTTCTTCCTTTTCTTCTTCC-3’, for fragment 1, upstream primer G8 5’- TAAAAAGGAAGAAGAAAAGGAAGA-3’ and downstream G9 reverse 5’-CAGAGGTACATCATTTGTATGA-3’ for fragment 2, upstream primer G 20 5’-AGTATGATGATAAGGATATTCAAGGAAAAA-3’ and downstream primer G 21 5’-CTCTATTATTTTCATCAAATAAAAG-3’ for fragment 3, upstream primer G18 5’-TTTAAAAATGAAGATCTTTTATTTGA-3’ and downstream primer G2 5’-TCATTTGATGCGTCCCTTAACC-3’(fragment 4), 25 mm MgSO_4_, 1 mM, AMV reverse transcriptase 0.1 U/μL) and a proof-reading ReproFast Taq polymerase (Genaxxon, Ulm, Germany) (0.1 UlμL) and 1.2 μg mRNA. PCR fragments were checked by submarine electrophoresis and sequenced.

### Assembly of the cDNA fragments

**Fig 1:**
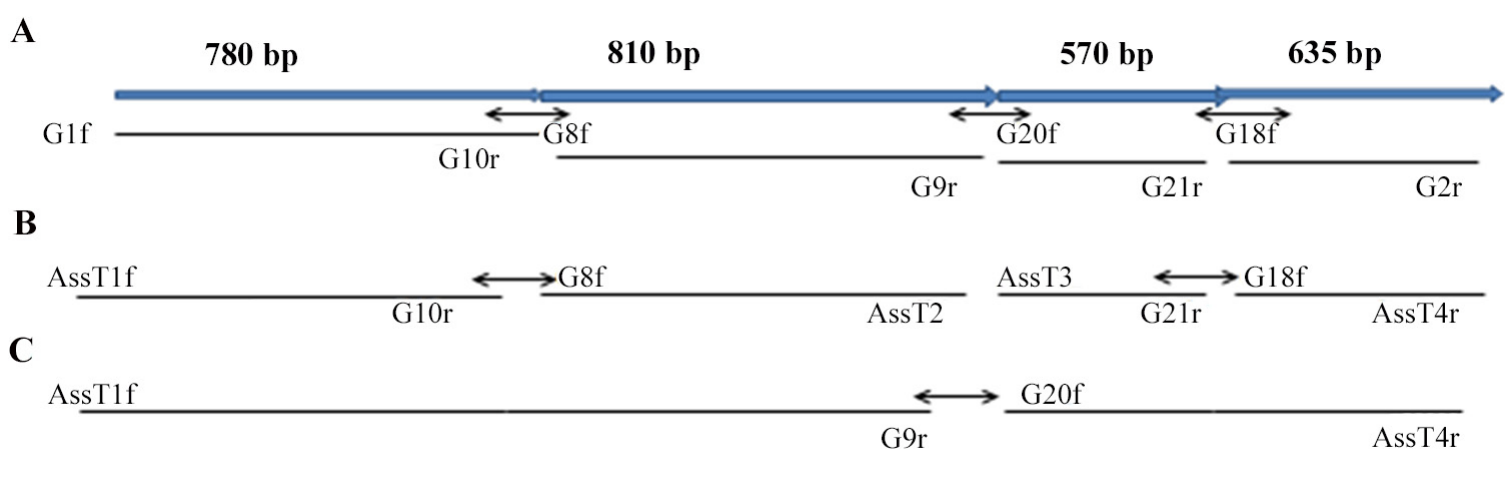
Cloning strategy of the nucleic acid sequence encoding the PfG protein from *Plasmodium falciparum*: A set of four different primer combinations was applied for the amplification to cover the complete coding region of PfG protein from *P*. *falciparum*. The primer pairs G1f# and G10#r, Gs 8f# and G9#r, G20f# and G21#r, G18f# and G2#r were employed in RT-PCR reactions with polyA^+^-RNA from schizont stages. Four different cDNA fragments in the range of 780 bp, 810 bp, 570 bp and 635 bp were obtained, respectively. Bold arrows in blue represent the full sequence of 2.7 kb while small black arrows with double arrowheads indicate the overlapping regions between the different fragments. Black lines represent the different cDNA fragments obtained with the sequence-specific set of primers used for the different amplificates. Downstream primers are located at the bottom of the black lines. B) Assembly reactions were performed with two fragments in each reaction from a total of four fragments. Primer combinations in particular assembly primers (assT1# and assT2#) with protruding 5’ and 3’ ends for subcloning into *pET-*28a expression vector were employed in the different PCR reactions (see Material and Methods within). From four amplificates two were assembled in each reaction with the primer set shown in the figure. C) Final assembly reaction of the two obtained fragments from reaction B resulted in the entire fragment of 2.7 kb.

For the assembly of DNA fragments the protocol from Gibson was employed [29]. Double stranded DNA fragments with overlapping ends were assembled in a 1 hour incubation at 50°C using three enzyme specific activities, i.e. the exonuclease, polymerase and ligase activity. Moreover, sequence specific primers overlapping the vector sequence of *pET-28a* were employed (Fig 1): i) for fragments 1 and 2 primer combinations Ass T1 for# 5’-GCT CG GTG CGG CCG CAT GAA AAA TGT TTT AAA AAA-3’(36 bp) and AssT2rev# 5’-AAT TCC CCT CTA GAC AGA GGT ACA TCA TTT GTA TGA-3’ (36 bp) were applied ii) for fragments 3 and 4 AssT3for# 5’-GCT CGA GTG CGG CCG CAG TAT GAT GAT AAG GAT ATT C -3’ (36 bp) and AssT4 rev# 5'-ATGGGTCGCGGATCCTCATTTGATGCGTCCCTTAA-3' (35 bp) were used. For a protocol which was optimized for a 2–3 fragment assembly, the total amount of fragments in the reaction was 0.02–0.5 pmol supplemented with Gibson Assembly Master Mix in a 2-fold concentration (10 μl) and deionized water to a total of 20 μl. After the assembly reaction the mix was transformed into *E*. *coli* NEB 5-alpha competent cells and selection was performed on kanamycin resistant LB agar plates at 37°C. For the final assembly step of the (35 bp) two obtained fragments, the primer combination AssT1 for# 5'-GCTCGAGTGCGGCCGCATGAAAAATGTTTTAAAAAA-3' (37 bp) containing a *NotI* restriction site (underlined) and G9 rev# 5’-GAT TTT CTT GAA TAT CCT TAT CAT-3’ (24 bp),G20 # for 5’-AGT ATG ATG ATA AGG ATA TTC AAG AAA A-3’ (28 bp) and AssT4# for 5'-ATGGGTCGCGGATCCTCATTTGATGCGTCCCTTAA-3' reverse (35 bp) (containing a *BamHI* restriction site) together with 64ng of the *NotI/BamHI* double digested *pet28a* vector were applied. All constructs were substracted to sequencing.

### Expression of recombinant PFG protein in *E*.*coli* and its native purification by nickel chelate affinity chromatography

For expression experiments the recombinant PfG plasmid was transformed into *E*.*coli* Rosetta cells. Overnight cultures of 100 mL LB medium which were supplemented with 30 μg kanamycin per mL were employed. 25 mL of fresh LB medium containing kanamycin was added the next morning to the culture. After 1 h of shaking 400 μL IPTG (100mM) was added and induction was performed for 3 h. Cells were harvested in aliquots of 25 mL and centrifuged at 4°C at 4000 rpm. For protein purification of the histidine-tagged PfG the pellet of 25mL culture was lysed with Bug Buster (Life Technologies, Darmstadt, Germany) and 1,5 μL Benzoanase (3Units/mLculture) (Merck, Darmstadt, Germany) for 20 min at RT. Subsequently centrifugation for 20 min at 12500 rpm, 4°C followed. The supernatant was subtracted to native protein purification according to a modified protocol from Qiagen, Hilden, Germany. Ni-NTA spin columns were equilibrated in 600 μL NPI-1 buffer containing 1mM imidazole. Column washes were performed in NPI-5 buffer with 5 mM imidazole. For the elution of the purified protein NPI-500 buffer with an imidazole concentration of 500 mM was employed.

### Native Blue PAGE gel electrophoresis

Proteins were separated on 4–16% Native PAGE^TM^ Novex Bis-Tris-Gels (Life Technologies, Darmstadt) by Native Blue PAGE electrophoresis according to a protocol from Life Technologies, Darmstadt. Samples were prepared in Native PAGE Sample buffer (4x) and separated in Native PAGE Running buffer (1x) and cathode buffer additive (1x). Staining was performed with colloid blue (Roth) for 15 h at room temperature (RT). A subsequent destaining was performed several times with a wash solution of 25 mL methanol and 75 mL H_2_O.

### Immunodetection

Immunoblotting was performed to detect the histidine-tagged PfG. 3 μg/μL mouse monoclonal penta-his antibody (Qiagen, Hilden) in TBS buffer with 3% BSA was applied for detection. The histidine-tagged PfG which was purified under native conditions was separated on a native 3–12% PAGE Novex Bis-Tris gel and purified protein fractions were blotted onto a PVDF membrane according to a protocol for western blotting of native 3–12% PAGE Novex Bis-Tris gels (Life Technologies, Darmstadt) using the iBlot Dry Blotting System from Invitrogen (Life Technologies, Darmstadt). Program P3 and a 7 min transfer time was applied. Gels were incubated with shaking in 2x NuPAGE transfer buffer prior to transfer. After the transfer, membranes were washed for 5 min in 8% acetic acid. The membrane was kept in blocking buffer (TBS buffer with 3% BSA) for at least 60 min, washed twice with TBST-buffer and incubated with the monoclonal penta-histidine antibody (300 ng/μl) (see immunodetection within). After washing with TBST buffer the primary anti-his monoclonal antibody was detected with anti-goat IgG Biotin antibody in a dilution of 1: 20.000 for 1 h. Subsequenty the membrane was incubated in a dilution of Extravidin-alkaline phosphatase (1:100.000) (Sigma-Aldrich, Munich) before it was developed in BCIP-NBT solution (Sigma Aldrich/ Munich). Alternatively, detection was performed with an IRDye 680 R labeled secondary anti-mouse IgG antibody which was diluted 1:20.000 fold. Incubation was performed for 50 min at room temperature in PBS buffer containing 5% skim milk powder and 0.2% Tween 20. Blots were washed three times with PBS buffer containing 0,1% Tween 20 for 5 min each and incubated 2 hours in the dark. Visualization of immunoblots were performed using a LI-COR Odyssey fluorescence system. Immunodetection by silver staining was performed according to a protocol by Roth (Karlsruhe, Germany).

### BODIPY GTPγS binding assay

For binding assays BODIPY® FL GTPγS analog [30] was employed to determine the specific binding activity of the GTP-binding protein. Fluorescense was monitored for samples of 50 nM BODIPY® FL GTPγS and 200 nmol protein in water. Fluorescense exitation (470 nm) and emission (510 nm) were determined in a fluorescense microplate reader (GoMax-Multi, Promega, Karlsruhe).

### GTPase activity

Determination of GTPase activity was based on a colorimetric assay kit according to a protocol from Innova Biosciences (Hamburg, Germany) with Pi-free GTP to prevent background signals. The released Pi formed a complex with a coloured green malachite dye that showed absorption in the wavelength range of 590–660 nm. Enzyme activity was performed using the equation Activity = (AC) /500 B where A is the concentration of Pi, C is the reciprocal value of the enzyme dilution factor and B is the assay time in minutes. One unit is defined as the amount of enzyme that catalyzes the reaction of 1μM substrate per minute.

### Kinetics of GTP binding

For the characterization of the kinetic parameters, purified enzyme preparations of *PfG* from *P*. *falciparum* and the stimulatory, constituitively expressed G-α subunit from human were applied. At t = 0 s the purified enzyme preparations of the G-proteins were mixed with 50 nM BODIPY® FL GTPγS and monitored for 600 s in a Perkin Elmer LS45 fluorescence spectrometer. The plot of product fluorescence versus time was fit linearly to obtain the product formation rate in relative fluorescence units (RFU)/s for each substrate concentration. Fluorescense values were monitored on the basis of a standard curve with defined substrate concentrations (Supplementary File). The determined values were corrected by background of spontaneous decomposition of BODIPY® FL GTPγS to BODIPY® FL GDPγS.

### Constitutively expressed human G protein alpha s long subunit

For control experiments the human G-protein alpha s long subunit with the Q227L mutation (GNA0SL00000) was employed which was purchased from Missouri S&T cDNA Resource Center, Missouri, United States. The mutation was performed by the Quickchange mutagenesis kit (Stratagene). The mutated open reading frame was cloned into pcDNA3.1+ (Invitrogen) at the KpnI (5') and Xho I (3') sites and amplified by the PCR from IMAGE clone (2448174). The mutation reduces GTPase activity resulting in a constitutively active phenotype. The insert size was 1195 bp.

### Peptide antibody against the PFG protein

A peptide antibody against the PFG protein was designed employing the peptide sequence NENNENVKNENVK (GenScript, New York). For conjugation to Keyhole Limpet Hemocyanin an extra C was added to the N-terminus. The antigen affinity purified antibody was employed in a dilution of 1:1000.

### Immunoprecipitation

Proteins from trophozoites and schizonts enriched fractions were solubilized with RIPA Buffer (Life Technologies, Darmstadt). Immunoprecipation was performed employing SureBeads ^TM^ Protein G Magntic Beads (Biorad, Munich). The eluates were checked on SDS PAGE and in parallel by western blot analysis as previously described under immunodetection within.

### Immunolocalization

*P*. *falciparum* (3D7 clone) cultures were maintained according to standard protocols. Mixed stage cultures [31] were fixed in 4% paraformaldehyde/0.0075% glutaraldehyde and processed for immunofluorescence as previously described [32,33]. Anti-PfG antiserum was used at a dilution of 1/1000, secondary antibodies (Dianova) at 1/2000. Images were taken on a Zeiss Cell Observer system using appropriate filter sets. Raw image data was processed using ImageJ. Images are representative of at least 20 independent observations.

## Results and Discussion

### Molecular Cloning of the PfG encoding nucleic acid sequence and determination of its stage-specific transcript levels

**Fig 2:**
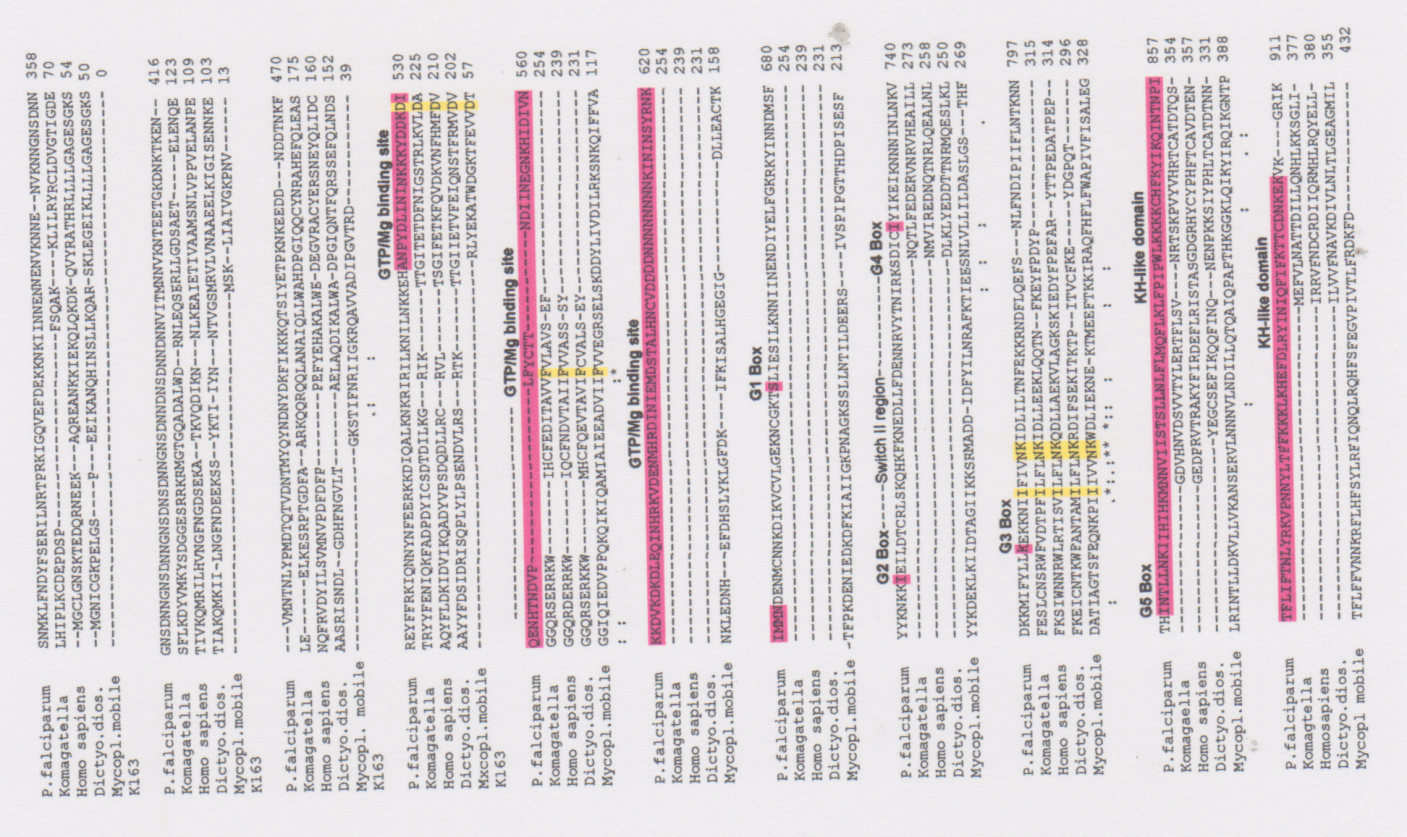
Multiple amino acid alignment of a stimulatory Gα_s_-subunit from *human* (G_s_αlpha-subunit), *Dictyostelium dioscoidum*, *Komagatella pastoris* (*Pichia pastoris*) and the amino acid sequence of the PfG protein spanning amino acid sequence positions from 361–911 for a better alignment. The EngA protein from *Mycoplasma* mobile strain K163 was included. Conserved amino acids present in the EngA2 domain are marked in yellow. This EngA2 subfamily represents the second GTPase domain of EngA and its orthologs, which are composed of two adjacent GTPase domains. Since the sequences of the two domains are more similar to each other than to other GTPases, it is likely that an ancient gene duplication, rather than a fusion of evolutionarily distinct GTPases gave rise to this family. Although the exact function of these proteins has not been elucidated. Asterisks label amino acid identities, colons (:) and dots (.) label amino acid similarities. The alignment represents the putitative characteristic domains of the PfG protein from *Plasmodium*. At the C-terminus a KH-like domain (purple colour, amino acid position 805–875) of the EngA subfamily of essential bacterial GTPases with two adjacent GTPase domains is located. The EngA2 domain covers amino acid positions 730–800. The GTPase domain elements for binding ribonucleoproteins are missing. A small GTP-binding domain respresents a Ras-like protein which is similar to the DER protein in *E*. *coli* responsible for cell viability and also present in *Neisseria gonorrhea* and *Thermotoga maritima*. This EngA2 subfamily CD represents the second GTPase domain of EngA and its orthologs, which are composed of two adjacent GTPase domains. Since the sequences of the two domains are more similar to each other than to other GTPases, it is likely that an ancient gene duplication, rather than a fusion of evolutionarily distinct GTPases, gave rise to this family. Although the exact function of these proteins has not been elucidated. The GTP/Mg-binding site is marked by a blue arrow and responsible for the chemical binding of GTP and Mg^2+^. Five G Box motifs for GTP-binding are present within the EngA2 domain including a switch region II which is a surface loop undergoing conformational change upon GTP-binding. The G1 box motif: GXXXXGK[T/S] is a signature motif of a phosphate-binding loop. G2 box motif: T Thr is conserved throughout the superfamily, but surrounding residues are conserved within families. G3 box motif: DXXG overlaps the Switch II region, which includes the Walker B motif. G4 box has the motif: [N/T]KXD and the G5 box the motif: [C/S]A[K/L/T].

Based on a bioinformatics screen with conserved amino acid sequences of the G-protein alpha-subunit from various organisms i.e. *Dictyostelium dioscoideum*, *Mycoplasma*, and *Saccharomyces cerevisiae* we identified an open reading frame (ORF) of 912 amino acids encoding a putative GTP-binding protein (*PFG*) present in the *Plasmodium* database (PlasmoDB identifier PF3D7_ 0313500). Total cellular RNA was isolated from schizont stages of *P*. *falciparum in vitro* and mRNA was employed. Poly (A^+^) m-RNA was applied in Reverse Transcriptase (RT)-PCR reactions with gene-specific primers (see Experimental section within). Fig 1 illustrates the workflow of the cloning procedure. The first cDNA fragment was identified with the combination of primers G6# and G7# and subsequent, nested PCR amplification employing primers G#8f and G#9r resulting in an amplificate of approximately 810 bp (fragment 2 (Fig 1). This fragment showed 100% nucleic acid identity to PlasmoDB identifier PF3D7_ 0313500 encoding a part of the putative G-binding protein (Fig 2). Moreover, the PCR amplificate showed significant amino acid identity scores of 72% on the amino acid level to the EngA domain [34] of different prokaryotes. EngA proteins are a unique family of bacterial Ras GTPases with two tandem GTP binding domains and a KH-like domain [35] which is depicted in Fig 2.

Next, two primers i.e. G1f# and G10r# overlapping at the 5’ prime and 3’ prime end of the obtained fragment 2 were designed. After RT-PCR, an amplificate of 780 bp was obtained covering the N-terminus of the protein (fragment 1). Fig 1 illustrates the workflow of the cloning procedure presented by primers and amplified fragments on top of the amino acid sequence. A similar strategy was applied with primers G#20f and G#21r designed with overlaps to extend the 810 bp fragment downstream (fragment 2). This amplification step resulted in a 570 bp fragment (fragment 3). Fragment 4 was amplified with primers G#20f and Ass#4overlapping the 5’ end of fragment 3. After sequence confirmation of the four fragments the assembly reaction was performed. In a first set of experiments fragment 1 and fragment 2 were combined. Subsequently, fragment 3 and fragment 4 were assembled (Fig 1). The successful reaction resulted in two fragments with the expected size of 1590 bp and 1255 bp, respectively. These fragments were resequenced and subsequently assembled to the complete fragment of 2736 bp, encoding the whole open reading frame (Accession No. HE667736, EMBL DATABASE) (Fig 1). The ORF of *PfG* was subcloned in p*ET-28a* expression vector [36] and the obtained recombinant plasmid resequenced (MWG Eurofins, Munich, Germany).

*PfG* from *Plasmodium* is an AT rich gene with an AT content of 80%, significantly surpassing the GC content. The deduced amino acid sequence of putative *PfG* shows that it is widely spread among different *Plasmodium* species. Most notably, is the significant amino acid identity to the orthologues from the rodent malaria parasites *Plasmodium chabaudi*, *Plasmodium yoelii* and *Plasmodium berghei* ANKA strain with 72%, 56% and 73% amino acid identity, respectively. By contrast, there is less homology to the two orthologous genes from the human malaria parasites i. e. *P*. *knowlesi* and *P*. *vivax* with 46% and 44%. The CLUSTALW amino acid alignment (Fig 2) with the included amino acid sequence of the human G_s_ alpha subunit diverges significantly in its homology from PfG. Similar results were obtained in an amino acid alignment with the 19 different human G-alpha subunits (data not shown). *PfG* shows different matches to the EngA2 domain [34] present in the Ras-like GTpase superfamily (amino acid position 800–875) comprising five G Box motifs for nucleotide binding.The different G Box motifs are depicted in Fig 2. Hence, it is worthy to note that the G box 3 motif DXXG contains asparagine (D) which forms a water-bridged contact with Mg^2+^ while glycine (G) is hydrogen bonded to the gamma phosphate of GTP through the backbone amide.

**Fig 3:**
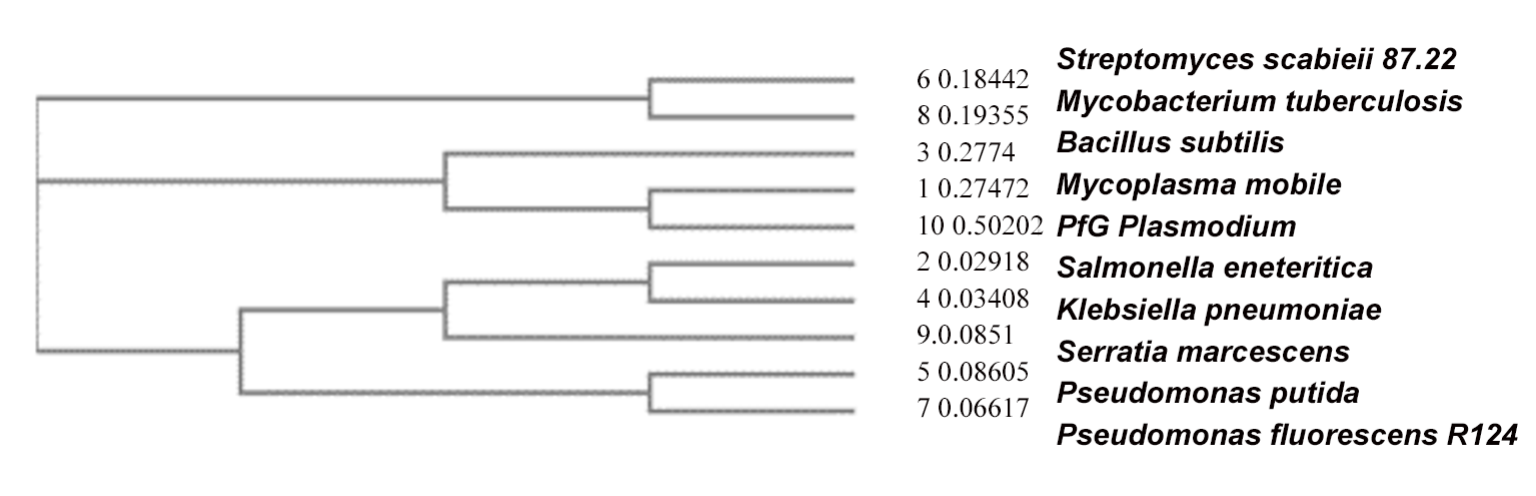
Phylogram pinpointing the phylogenetic origin of the EngA2 domain in the PfG protein from *Plasmodium*: The closest phylogenetic relationship is evident between *Plasmodium* (10) PfG and *Mycoplasma mobile* strain K173 (1) with a determined difference in tree distance of 0.245. Both are closely related to the EngA2 domain from *Bacillus subtilis* (3) with a difference in tree distance of 0.0027 in comparison to *Mycoplasma mobile* (1). The next closest relationship of the EngA domain is to *Streptomyces scabieii* (6) (tree difference:0.18442) and to *Mycobacterium tuberculosis* (8) (tree difference:0.19355) rather than the *Enterobacteriaceae Salmonella enteritica* (8) *and Klebsiella pneumonia* (4) *and Serratia marcescens* (9) with tree distances of 0.02918, 0.03408 and 0.02918, respectively. Less related are the EngA2 domains present in *Pseudomonas putida* (tree difference:0.086057) (5) and *Pseudomonas fluorescens* (tree difference:0.06617).

The obtained data prompted us to generate a phylogram to identify the closest phylogenetic relationship of the EngA domain present in PfG to other species.The phylogram depicted in Fig 3 shows a close relationship with a tree difference of 0.245 to *Mycoplasma mobile*, a pathogenic gram positive Eubacterium and to *Bacillus subtilis* with a tree difference of 0.0027, respectively. *Mycoplasma mobile* is one of the fastest smooth gliding bacteria. In sum, the PfG protein cannot be classified within mammalian Gα-subunits. In a search within the PlasmoDB database we were unable to discover a G-protein ß subunit. Instead, we identified a G-beta repeat domain containing protein (PFIT_-_1122200). There was no database entry encoding a G-protein gamma subunit. These results are in contrast to previous findings which demonstrated the occurrence of heterotrimeric G-proteins in *Plasmodium* by western blot analysis with cholera and pertussis toxins employing antibodies directed against epitopes of different human G-alpha subunits [37].

In parasitic protozoa only a few heterotrimeric G-proteins have been identified. A Blast screen of the PfG nucleic acid sequence showed no orthologues in other Apicomplexan parasites. A highly divergent Gα-subunit has been characterized in the Apicomplexan *Entamoeba histolytica* [38]. This parasite causes symptomatic amoebic colitis which leads to 100,000 deaths per year worldwide. The divergent G alpha-subunit from *Entamoeba histolytica* modulates cellular processes related to pathogenesis when expressed in trophozoites, in particular the enhanced host cell attachment kills the human host cells. Moreover, it was shown that the *Entamoeba histolytica* Gα-subunit exhibits nucleotide cycling properties although it is of early evolutionary origin apart from the metazoans. Hitherto, there are no more reports about the occurrence of heterotrimeric G-proteins in other Apicomplexan parasites. An overexpression screen in different Apicomplexans resulted in the identification of a basic set of small GTPases with high conservation over the whole lineage. Mostly represented are the small GTPases like Rab4 or Rab6. In *Toxoplasma gondii* RAB5A and RAB5C are involved in transport to secretory organelles which was proven by functional ablation of the genes [39] resulting in aberrant secretory transport to micronemes and rhoptries. As Apicomplexan parasites invade the host cells, virulence factors of micronemes are released under the control of GTPases pinpointing their role in infection [40].

Heterotrimeric G-proteins have been identified in the blood fluke *Schistosoma mansoni* [41] and the liver fluke *Fasciola hepatica* [41] and are part of the transmembrane-signaling system. Immunoblot analysis was performed to distinguish between the different subunits raised against peptides from the encoded bovine cDNAs. A G_αs_ subunit and a G_0α_ subunit in the range of 41 kDa and a Gß subunit with a size of 36 kDa were identified. Recent experiments demonstrated [42] the occurrence of a biogenic amine G-protein-coupled receptor from *Schistosoma mansoni* with 30% sequence homology with all major types of GPCRs. Significant differences appeared in the third domain where a highly conserved asparagine (Asn^111^) was substituted. However, this mutation did not have an impact on the responsiveness of the receptor to histamine.

**Fig 4:**
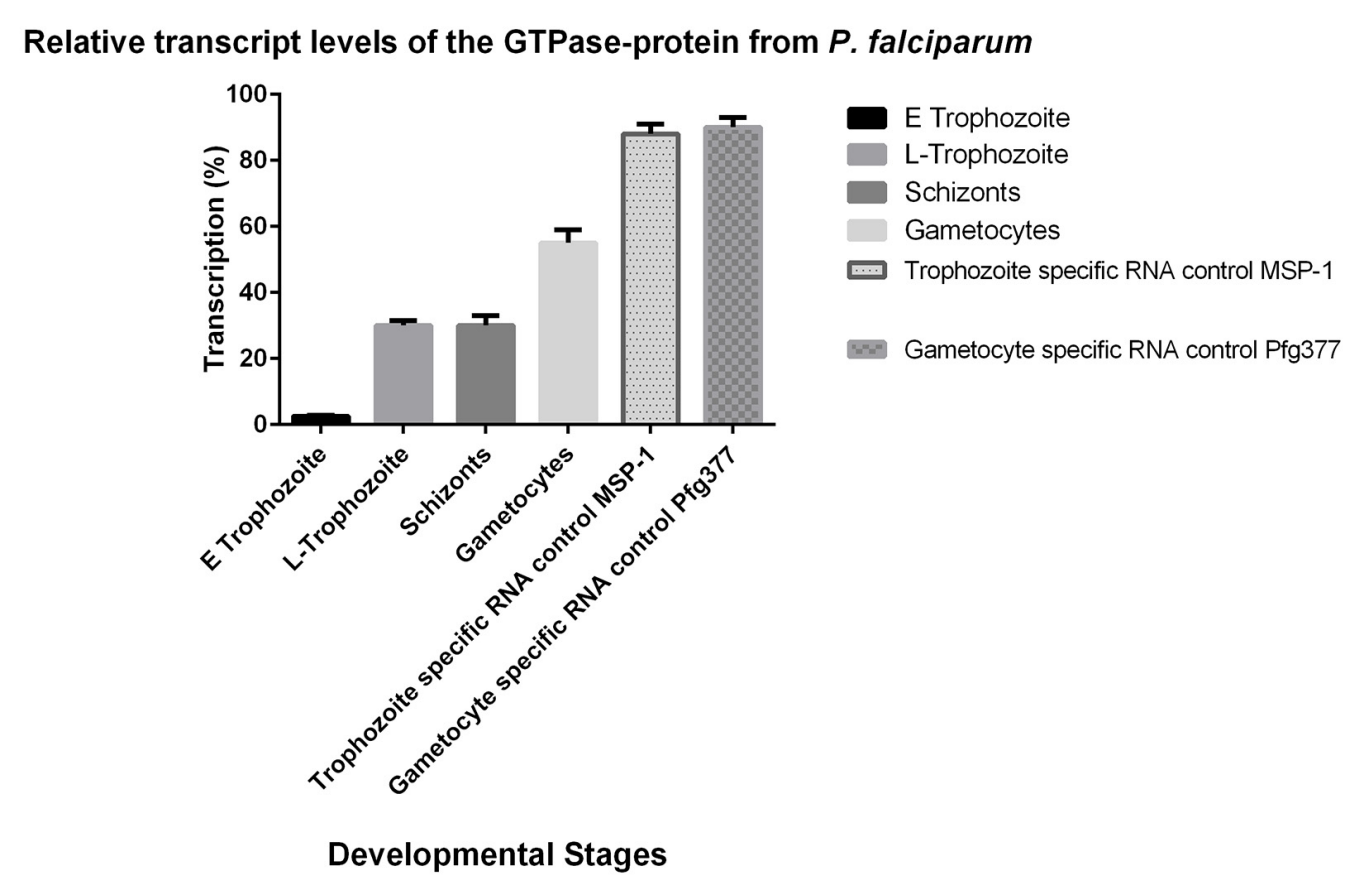
Transcription levels of the *PfG* mRNA from *P*. *falciparum*. Northern Blot analysis with cellular RNA from different developmental stages: E Trophozoite = Early Trophozoite; L Trophozoite = Late Trophozoite; Schizonts and Gametocytes. Detection of signals was performed by phoshoimage analysis using the digoxigenin–dUTP-labeled alpha-tubulin 2 gene as a constituitively expressed gene in the blood stages or the *PfG* cDNA from *P*. *falciparum* as molecular probe. Two stage specific controls were employed:i) The MSP1 fragment resulted in a signal of 5161bp in the blood stages and a signal of 3.8 kb in the gametocytes.

Next, we monitored transcription of the *PfG* m-RNA in different developmental stages i.e. young and late trophozoites, schizonts and gametocytes. (Fig 4). The alpha tubulin 2 gene [27] from *P*. *falciparum* (PF3D7_ 0903700) was employed as a constitutively transcribed control in RNA prepared from mixed erythrocytic stages [25]. Transcription of *PfG* increased approximately 12-fold in mature trophozoites and schizonts. This result prompted us to investigate transcript levels in sexual precursor stages like gametocytes where a 22-fold increase was detected. Stage specific probes were employed as controls, i. e. a 92 bp fragment from MSP-1 (merozoite surface protein1) transcribed in the erythrocytic stages and [28] and the 1223 bp probe from the Pfg377 *var* gene for gametocytes resulting in transcript levels of approximately 90% for both probes in each developmental stage (Fig 4).

### Expression, purification and functional characterization of PfG from *Plasmodium*

**Fig 5:**
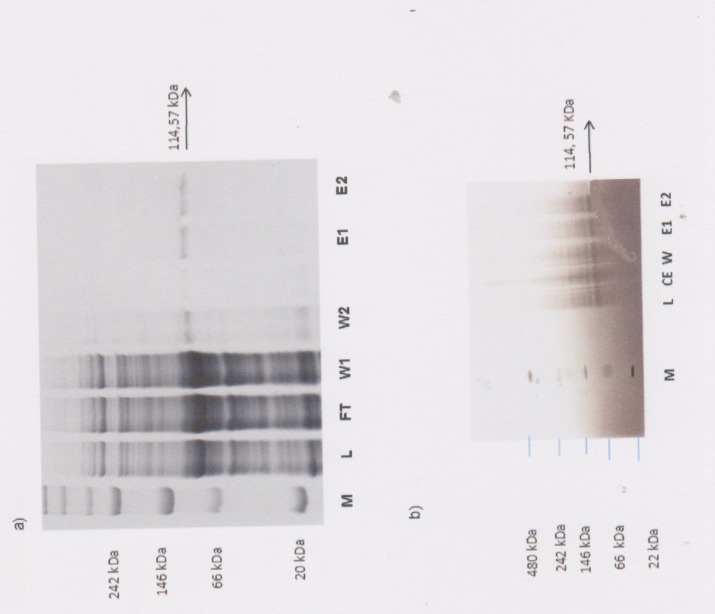
Native Blue Gel electrophoresis after expression and purification of the PfG protein under native conditions by nickel-chelate-chromatography. Separation was performed on a 6–12% Bis-Tris gel. a lane 1 Native unstained protein marker (NativeStain^TM^) 20kDa = Soybean Trypsin Inhibitor, 66 kDa = Bovine Serum Albumin, 146kDa = Lactate Dehydrgenase; 242 kDa = B-phycoerythrin, 480 kDa = Apoferritin band 2; lane 2 lysate; lane 3 flowthrough; lane 4 wash fraction 1, lane 5 wash fraction 2; lane 6 eluate 1; lane 7 eluate 2. b Immunoblot characterization of the different affinity purification steps of PfG protein from *P*. *falciparum*. Native-Blue-PAGE electrophoresis was employed to separate the purified proteins. Subsequent immunoblot analysis followed using a monoclonal anti MBP (a murine anti-maltose binding protein) His antibody.

**Fig 6:**
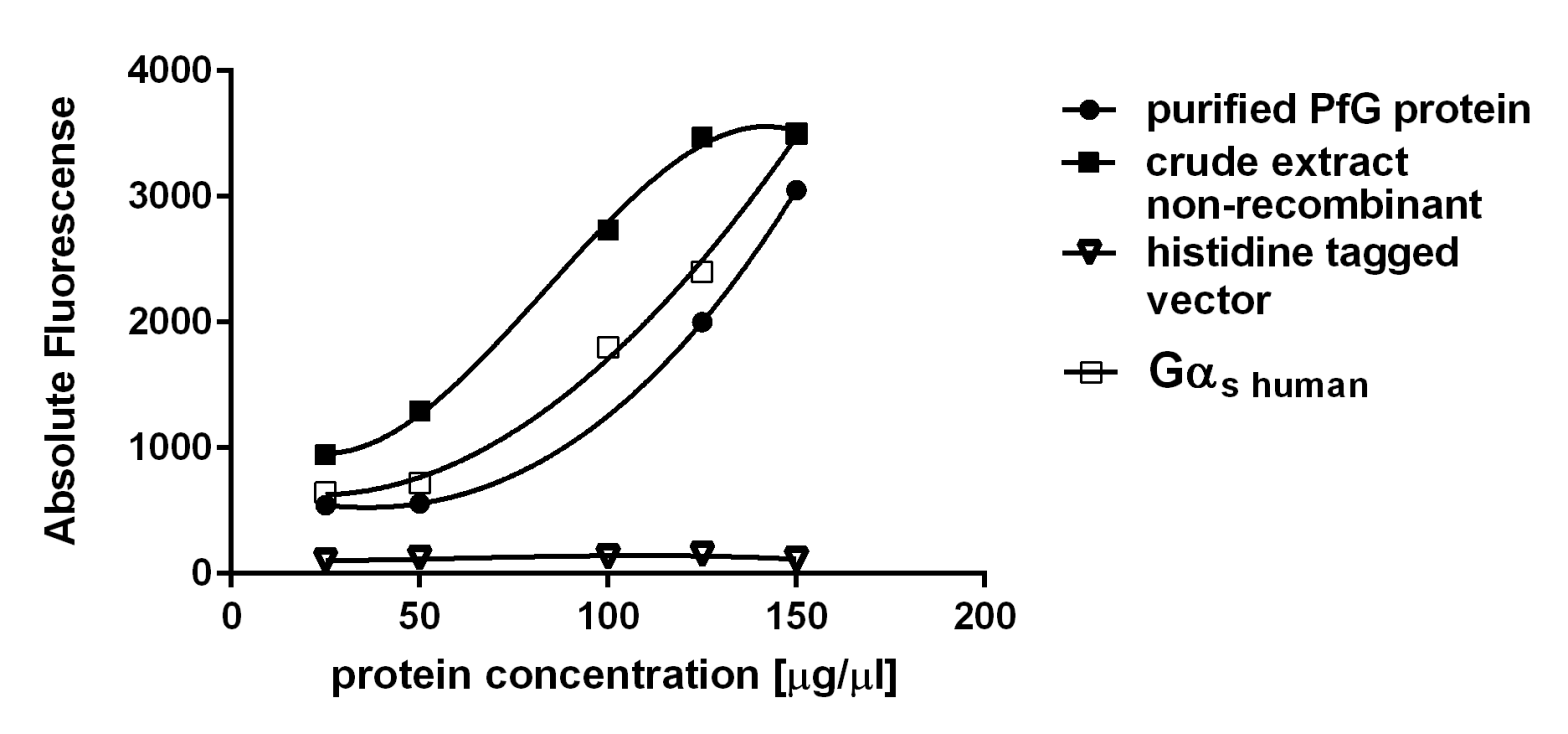
Saturation curve representing the binding of BODIPY®FL GTP_Ύ_S with the expressed PfG protein from *P*. *falciparum*. The depicted plot shows the absolute fluorescense.of the formed PfG-Protein-BGTP complex monitored at a wavelength between 480 nm and 510 nm versus increasing protein concentrations with a crude extract of the purified expressed PfG- protein (black square) and a purified enzyme preparation (black circle) after native purificaion. A non-recombinant *pET-28a* vector (open triangle) and a constitutively expressed Gα_s_ human subunit (open square) were employed as a positive and a negative control. Each point represents the mean value of three different experiments.

Next, *PfG* expression was performed in p*ET-28a* in *Escherichia coli* Rosetta cells harbouring the T7 RNA polymerase under the control of the IPTG-inducible T7 promotor [36]. Optimal yields (15 mg/L) were obtained 3 h after induction. Subsequent purification by nickel chelate affinity chromatography under native conditions showed a soluble protein present in both eluate fractions with a size of 108.57 kDa (Fig 5A). Detection of the protein was only possible after separation by Blue Native PAGE while separation under denaturing conditions caused misfolding of the PfG protein (data not shown). A monoclonal anti-mouse penta-histidine tag (Fig 4B) antibody diluted to a concentration of 3 μg clearly detected the protein with a molecular mass of 114,65 kDa (Fig 5B). The difference in the predicted molecular mass of 108,65 kDa of PfG and the detected, purified protein with a mass of 114,65 kDa is due to the presence of the six histidine residues which form the tag in the expression vector. BODIPY® FL GTP_Ύ_S was employed as a fluorescent probe to investigate binding to the protein since it is known to bind to the alpha-subunit of heterotrimeric G proteins yielding a significant increase in fluorescence emission. Increasing concentrations of native, purified PfG in the range from 27 μg to 130 μg were tested for binding of 25 nmol BODIPY® FL GTP_Ύ_S (Fig 6). Fluorescense emission constantly increased until a protein concentration of 110 μg was reached. Thereafter, saturation followed. In contrast to the purified extract, the crude extract showed significantly increased levels of fluorescence suggesting that compounds with unspecific fluorescence emission are present in the crude extract (Fig 5). These results were also obtained when lower protein concentrations of 10–40 μg were applied (data not shown). By contrast, a protein extract obtained from the non-recombinant p*ET-28a* vector which functioned as a control exhibited no significant binding of the BODIPY® FL GTP_Ύ_S (Fig 6).

Expression of recombinant, plasmodial PfG-protein in *E*.*coli* resulted in the protein of the correct size but with reduced stability to a maximum of 3.5 hours. Similar results have been reported for the aspartic protease plasmepsin, an enzyme of the hemoglobin-degrading pathway [43]. Different attempts for heterologous expression of the enzyme in a soluble, stable active form failed due to incomplete formation of disulfide linkages in the refolded enzyme under acidic conditions [43]. In case of the *P*. *falciparum* PfG no structural data are available to explain the instability of the protein. Hitherto, experiments to express the protein in *Nicotiana benthaminia* plants to improve its stability were only partly successful when the transmembrane domain was deleted (Kaiser et al., unpublished). A second alternative might be the expression after codon-harmonization in *E*.*coli* which was recently reported for the high level, stable expression of monofunctional S-adenosylmethionine decarboxylase [44]. Hitherto, we cannot exclude that PfG might be part of a multisubunit complex which might cause its instability.

Different screens in protein databases with the amino acid sequence of the PfG protein from *Plasmodium* resulted in significant matches to the EngA2 protein domain PF 01926 (Pfam), IP 52540 (Interpro) at amino acid positions 10–140 and 10–180 from different bacterial species, respectively. Most notably is the occurrence of the EngA2 domain in P-loop containing triphosphate hydrolases based on ß-sheet topologies [45]. In case of the universally conserved GTPase HflX from *Escherichia coli* the protein associates with the ribosomes [45]. Secondly, it has been recently demonstrated that GTPases are likely to play key roles in the assembly of ribosomes from bacteria and eukaryotes [45]. In this context it is tempting to speculate that PfG from *P*.*falciparum* might be involved in translational control since mammalian Ras proteins are involved in developmental processes like proliferation, cell fate and apoptosis [46]. However, it is unlikely that a ribonuclear protein is one of the interacting partners of the GTPase domain since typical binding motifs are missing.

**Fig 7:**
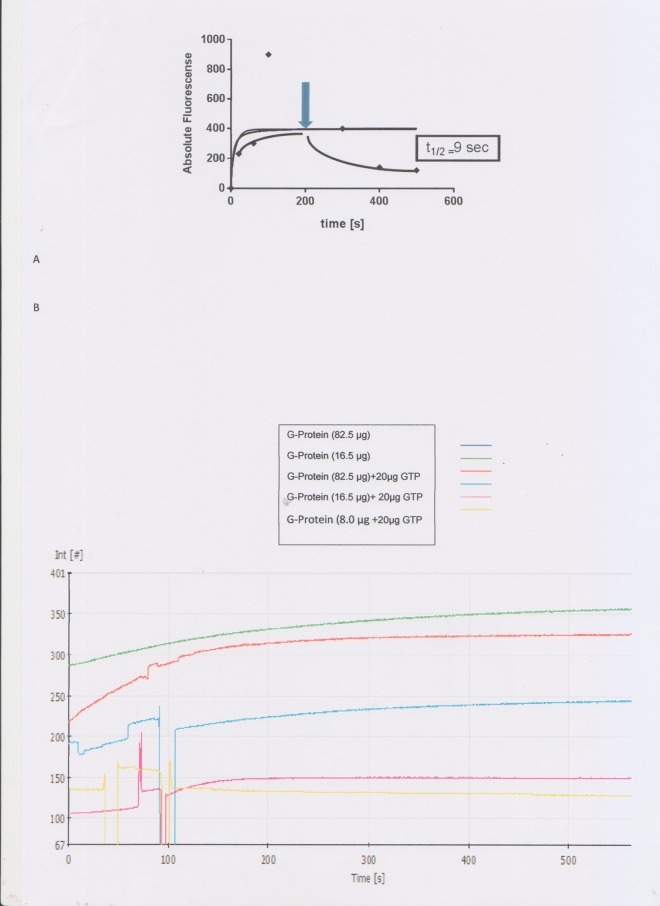
Association and dissociation of BODIPY FL GTPγS with PfG from *Plasmodium*. Part A Absolute Fluorescense of binding of 50 nmol BODIPY FL GTPγS to the plasmodial, PfG-protein (black line). The increase in fluorescence was monitored until saturation over a time interval of 600 s. Part B At the arrow at t = 200 s, 20 μM unlabeled GTPγS was added and the decrease in fluorescence was monitored. The dissociation of BODIPY FL GTPγS was fit with single exponential functions and the half life value was determined i.e. t_1/2_ = 9 s suggesting a low affinity for the binding of BODIPY FL GTP. b Monitoring GTP-binding of the *P*. *falciparum* G-protein in a time course experiment: Binding assay with different protein concentrations of PfG from *Plasmodium* green line 62.5 μg, red line 16,5 μg; the reaction was antagonized with unlabeled GTP after 100 s blue line (62,5 μg purified G-protein), purple line (16,5 μg purified-G-protein), yellow line (8,0 μg G-protein).

Association/dissociation was performed with 20 μM unlabeled GTP_Ύ_S. In a first experiment the unlabeled GTPγS was added at t = 0 s and an immediate decrease in fluorescence followed which was monitored in a Perkin Elmer LS45 spectrometer (Fig 7A). The absolute fluorescense value (180) was already lower after supplementation with 20μM unlabeled GTP after 0 s in comparison to 230 of the untreated control (Fig 7A). The unlabeled nucleotide competed directly with the fluorescense labeled GTP for the binding site of the PfG protein. In a second experiment the assay was supplemented with 20μM unlabeled GTP after 200 s and the exponential decay was monitored caused by dissociation of the labeled GTP from the PfG binding site (Fig 6A). By contrast, the control experiment showed a hyperbolic saturation curve without supplementation of unlabeled GTP (Fig 7A). This result is consistent with the determined half lives i.e.t_1/2_ = 71,14 s for the untreated control and the decreased half life t_1/2_ = 9 s for the antagonized reaction. Next, kinetic parameters of the reaction were determined. In this experiment the binding of BODIPY®FL GTP_Ύ_S to PfG was monitored over 600s under different substrate concentrations (Fig 6B). PfG had a determined Km for BODIPY®FL GTP_Ύ_S of 1.77 ± 0.36 nmol. By contrast, the human Gα_0_ and G_αi1_ subunits had a Km of 14±8 nmol and 87±22 nmol, respectively, demonstrating a high affinity for the nucleotide which is typical for a heterotrimeric G protein. Commitment to and completion of sexual development are essential for malaria parasites. It has been recently shown that a cascade of DNA-binding proteins is responsible for sexual commitment and development in *Plasmodium* [47]. These DNA-binding proteins belong to a group of transcription factors which are highly conserved in apicomplexan protists and amenable to environmental sensing. It will be an important issue in the future to delineate the function of the PfG protein in this context.

### Costimulation of nucleotide binding in the presence of Mastoparan and sodium fluoride

**Fig 8:**
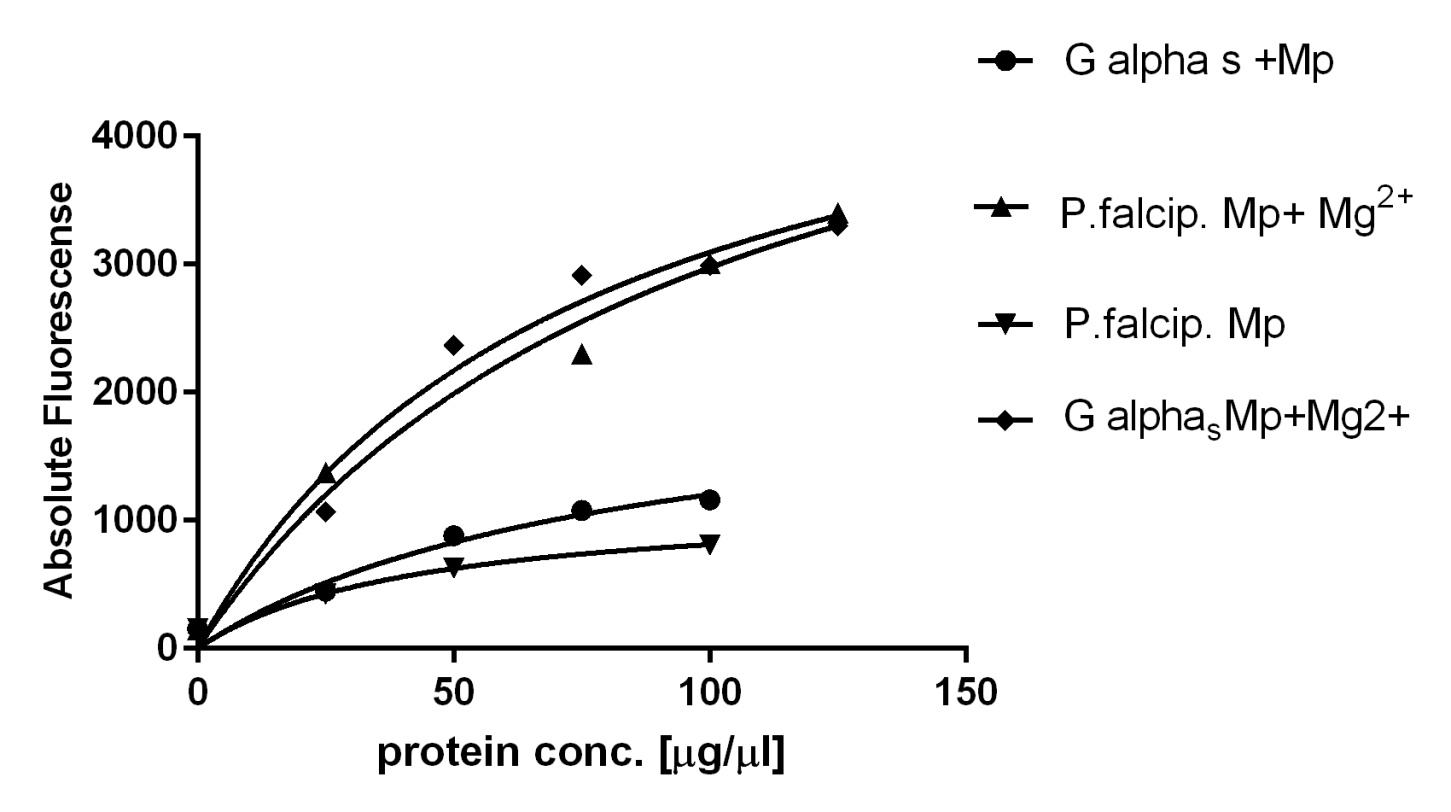
Costimulation of the constitutively expressed human G protein alpha_s_ long subunit in comparison to PfG from *P*. *falciparum*. The expressed recombinant proteins were stimulated with mastoparan alone simulating the receptor in case of Gα_s_ human (black circle) and PfG from *P*. *falciparum* (rhomb.) Costimulation was performed with mastoparan and Mg^2+^ for plasmodial PfG (triangle) and the constitutively expressed G alpha-s subunit (rhomb).

In the next experiments Mastoparan (Mp) [48] and sodium fluoride [49] known as stimulators of guanine nucleotide exchange were employed (Fig 7). While Mastoparan mimics the GPCR mode of action, the mechanism of sodium fluoride is based on the formation of a fluoroaluminate complex which can pass the membrane and occupy the position next to bound GDP and thus stimulates an active state conformation [49]. Based on these observations we first investigated the effect of sodium fluoride at different concentrations in the range between 10 to 100 μM. However, neither the constitutively expressed Galpha_s_ subunit from human nor the G-protein from *P*. *falciparum* showed a significant increase in binding of BODIPY®FL GTP_Ύ_S in the range of the tested sodium fluoride concentrations.This result prompted us to test binding of the fluorescence labeled GTP_Ύ_S under stimulation with Mp (Fig 8). While stimulation with Mp resulted in no significantly increased nucleotide exchange for the constitutively expressed Gα_s_ subunit from human and the expressed PfG protein from *Plasmodium*, costimulation with MgCl_2_ increased GTP binding approximately 4.5- fold for both proteins (Fig 8).

### Determination of GTPase activity

**Fig 9:**
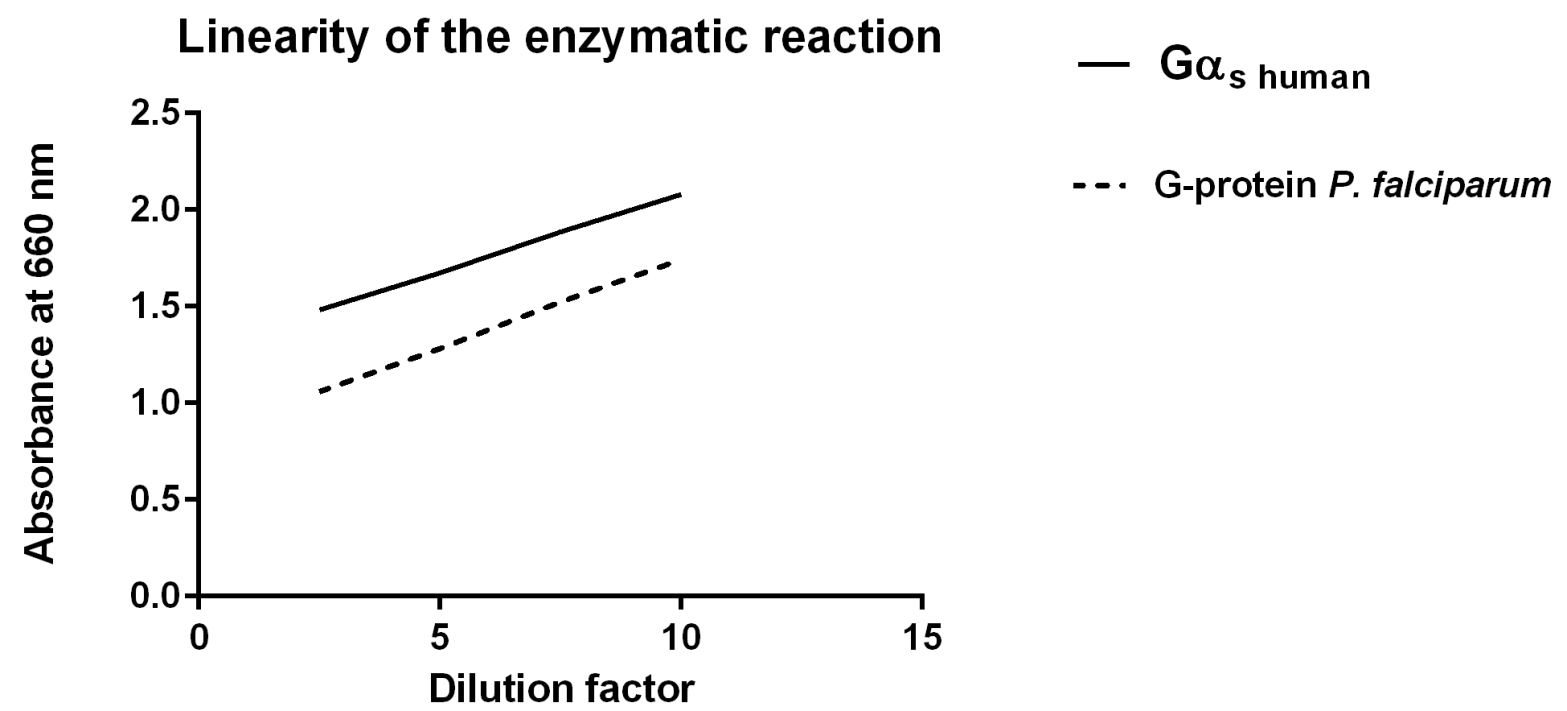
Absorbance versus enzyme amount: For quantification of the reaction absorbance versus enzyme concentration was plotted. To keep linearity, the assay time was fixed for 30 min and the temperature kept at 25°C.

The determination of GTPase activity was based on a colorimetric assay employing P_i_-free GTP and a malachite green formulation for complex formation with released P_i_. The malachite green formulation contained additives to prevent background signals from non-enzymatic GTP-hydrolysis [50]. Absorption was measured at 620 nm. In a first experimental step P_i_ contamination was checked in the purified enzyme preparation and in parallel in an assay without enzyme but with supplemented P_i_ at a concentration of 0.1mM. The purified enzyme preparation had an absorbance vaIue of <0.20 like the control without enzyme. The P_I_ containing probe showed an absorbance unit > 1.0. In a second step absorbance versus the amount of enzyme concentration was monitored (Fig 9). The results showed that the assay is linear up to OD_620_ of 1.8 in case of the human Galpha_s_ subunit and to an OD_620_ of 1.4 for PfG. In sum, a dilution factor of 5 was ideal for both enzyme preparations in the assay. Thereafter, absorption for the complete assays was measured starting with dilutions in the range of 2,5, 5 and 6 of the purified enzyme preparations. The released P_i_ in the different assays was determined employing a standard curve with different P_i_ concentrations. The determined GTPase activity was 0.6 x 10^−3^ units for *P*. *falciparum* G-protein and 50% less than the human Gα_s_ with 1x10^-3^ U (see unit definition within Experimental). These data demonstrated that PfG from *Plasmodium* has Ras-like GTPase properties rather than kinetic characteristics of a canonical G-protein: i) Although the determined Km value for BODIPY FL GTPγS 1.77 ± 0.36 nmol is in the nanomol range, the affinity for binding a fluorophoric GTP analogue is low compared to the human Gα_0_ and G_αi1_ subunits with a Km of 14±8 nmol and 87±22 nmol, respectively ii) PfG from *Plasmodium* hydrolyses GTP rather quickly. PfG had a determined half life of t_1/2_ = 9 s in comparison to the G_α0_-subunit from human with a half life of t_1/2_ = 1400 s [50]. These results contradict a canonical G-domain structure for PfG due to the fast dissociation of BODIPY FL GTPγS after addition of the unlabeled GTP.

### Detection of endogenous PfG in the erythrocytic stages

**Fig 10:**
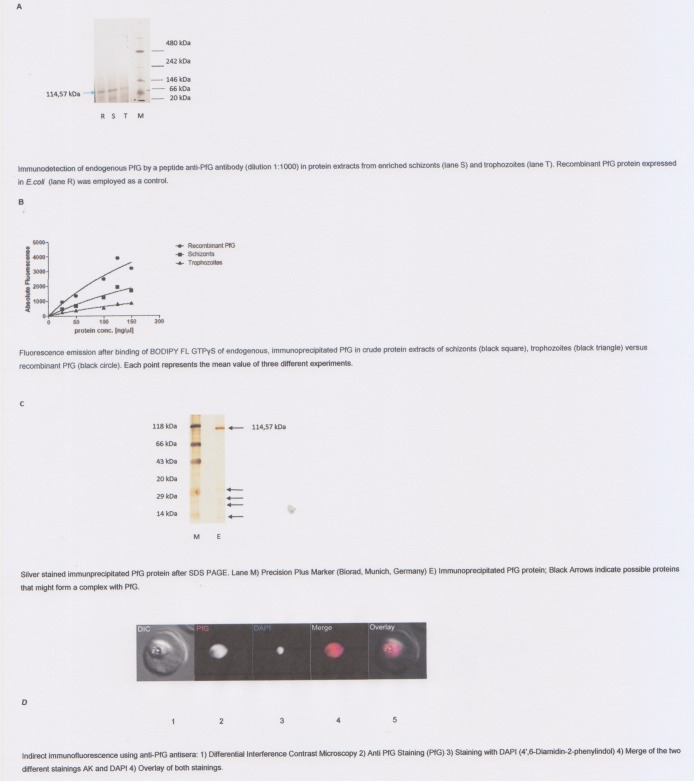
(a) Detection of endogenous PfG in the erythrocytic stages: A) Immunodetection of endogenous PfG by a peptide anti-PfG antibody (dilution 1:1000) in protein extracts from enriched schizonts (lane S) and trophozoites (lane T). Recombinant PfG protein expressed in *E*.*coli* (lane R) was employed as a control. (b) Fluorescence emission after binding of BODIPY FL GTPγS of endogenous, immunoprecipitated PfG in crude protein extracts of schizonts (black square), trophozoites (black triangle) versus recombinant PfG (black circle). Each point represents the mean value of three different experiments. (c) Silver stained immunprecipitated PfG protein after SDS PAGE. Lane M) Precision Plus Marker (Biorad, Munich, Germany) E) Immunoprecipitated PfG protein; Black Arrows indicate possible proteins that might form a complex with PfG. (d) Indirect immunofluorescence using anti-PfG antisera: 1) Differential Interference Contrast Microscopy 2) Anti PfG Staining (PfG) 3) Staining with DAPI (4′,6-Diamidin-2-phenylindol) 4) Merge of the two different stainings AK and DAPI 4) Overlay of both stainings.

To investigate the expression of endogenous PfG in the erythrocytic stages an anti-PfG peptide antibody was employed in crude protein extracts obtained from enriched trophozoite and schizont fractions (Fig 10A). The determined protein concentrations in 200 μL crude cell lysate were 150 μg for the trophozoites and 170 μg for the schizont fractions. The most significant expression was detected in the purified schizont fraction (S) and in the fraction of the purified, histidine tagged recombinant protein fraction (R). Endogenous PfG was also detectable in the trophozoite fraction (T), however with weaker signal intensity. Next, the functional activity of the endogenous, immunoprecipitated protein was tested (Fig 10B) for GTP binding. Fluorescence emission was significantly lower in schizonts (S) and trophozoites (T) compared to the recombinant PfG protein. Hence, it is remarkable that fluorescence emission was higher in protein extracts from schizonts than in protein extracts from trophozoites suggesting its developmental stage-specific dependence. Western Blot analysis performed with pre-immune serum resulted in no significant background signal (Kaiser, unpublished results).

Next, we tested complex formation of the immunoprecipitated PfG protein after SDS-PAGE by silver staining. For this experiment an eluate fraction obtained by affinity chromatography was employed (Fig 10C). Beside the significant band of 114,57 kDa a set of smaller bands with a size of 14kDa to 29 kDa was detected.To determine whether these proteins are subunits of a multisubunit complex or interacting proteins they have to be analyzed by mass spectrometry after tryptic digestion.

We were interested in determining the sub-cellular localisation of PfG. To this end we carried out an indirect immunofluorescence assay, using the specific antisera we had generated. Paraformaldehyde/glutardehyde fixed cells, labeled with our antisera, showed fluorescence associating only with the body of the parasite (Fig 10D). Pre-immune serum and secondary antibody only controls showed no specific fluorescence, and similar results were seen when using an alternate fixing method (data not shown), suggesting that this staining pattern represents the true localization of the endogenous protein. This localization to the parasite cytosol is consistent with the lack of any known protein targeting motifs within the primary protein sequence, and the lack of any hydrophobic domains which may act as membrane anchors.

Heterotrimeric G-protein signaling has provided a variety of targets amenable to pharmacologic manipulation. However, the most prevalent target is the GPCR itself [51]. In *P*. *falciparum*, the causative agent of malaria, elucidation of a GPCR- coupled pathway has just begun and may contribute to the discovery of alternative targets and improvement of antimalarial therapy. Most of the currently available antimalarial drugs do not eradicate the gametocytes, the sexual precursor stages of the parasites except the clinically applied primaquine. The recently discovered imidazopyrazines are promising novel lead compounds blocking a lipid kinase, the phosphosphatidylinositol-4-OH kinase the (PI4K) which phoshorylates lipids to regulate intracellular signaling and trafficking [52].

Here, we report the first identification of a non-canonical PfG protein, a Ras-like GTPase protein from *Plasmodium* resulting from a screening approach due to the absence of heterotrimeric G-proteins in *Plasmodium*.

Our results provide novel insights into malaria infection. Apart from the current opinion that the parasite exclusively depends on the host G_s_ for the invasion into the human erythrocyte, a parasitic PfG protein is present with significant, relative transcription levels in the sexual precursor stages but with still unknown function. A loss of function mutant will elucidate its role in stage conversion from the asexual to the sexual stages and its role in pathogenesis. Currently, an intensified screening is under way to identify a putitative GPCR [53,54] as an element of a non-canonical GTPase coupled signal transduction pathway in *Plasmodium*.
